# Screening for inborn errors of metabolism among newborns with metabolic disturbance and/or neurological manifestations without determined cause

**DOI:** 10.1590/S1516-31802001000500002

**Published:** 2001-09-01

**Authors:** Allan Chiaratti de Oliveira, Amélia Miyashiro Nunes dos Santos, Ana Maria Martins, Vânia D'Almeida

**Keywords:** Inborn Errors of Metabolism, Inherited Metabolic Disorders, Newborns, Urinary Screening, Erros inatos do metabolismo, Distúrbios metabólicos hereditários, Recém-nascidos, Triagem urinária

## Abstract

**CONTEXT:**

Inborn Errors of Metabolism are hereditary affections resulting from incompetence in enzymatic reactions of intermediary metabolism. At present, several hundred hereditary metabolic disturbances are known, many of which correspond to severe life-threatening disorders.

**OBJECTIVE:**

The early detection of carriers has motivated the screening for these disturbances among newborns at the Neonatal Unit of Hospital São Paulo, in an attempt to initiate support treatment, when available, before clinical manifestations become evident.

**DESIGN:**

Prospective study of risk patients.

**SETTING:**

Laboratory for Inborn Errors of Metabolism at the Center for Medical Genetics of the Departments of Pediatrics and Morphology of Universidade Federal de São Paulo/Escola Paulista de Medicina. Newborn care unit at a tertiary care hospital.

**PARTICIPANTS:**

101 children admitted into the Neonatal Unit were included in this study by presenting hypoglycemia, metabolic acidosis, jaundice, difficulty in gaining weight, diarrhea, vomiting, hepato- and/or splenomegaly, cataracts, apnea, convulsions, hypo- or hypertonia.

**DIAGNOSTIC TESTS:**

Tests routinely utilized, performed for qualitative research of abnormal substances excreted in the urine in situations of metabolic disorder.

**RESULTS:**

Children were included in the study mainly because of presenting hypoglycemia, jaundice and metabolic acidosis. Sixty-four newborns presented at least one positive test result. Most of the positivity was due to transitory metabolic alterations of the newborn, such as the case of Transitory Neonatal Tyrosinemia, presented by 29 patients. Nine infants were referred to the Center for Medical Genetics of Universidade Federal de São Paulo for continuation of the diagnostic investigation. For three of them, the tests applied permitted us to formulate a diagnostic hypothesis of mucopolysaccharidosis, tyrosinemia type I and non-ketotic hyperglycinemia, respectively.

**CONCLUSIONS:**

The high positivity observed in the tests reflects the newborn's own metabolic immaturity. The selection of 9% of the studied cases for outpatient follow-up confirms that Inborn Errors of Metabolism must be suspected whenever a patient presents metabolic disturbances or neurological manifestations without a determined cause. They should be researched in parallel with the other diagnostic possibilities and not just taken to be exceptional diagnoses.

## RESUMO

**CONTEXTO:**

Os erros inatos do metabolismo são afecções hereditárias resultantes da incompetência de reações do metabolismo intermediário. Atualmente já são conhecidos centenas de distúrbios metabólicos hereditários, muitos deles correspondendo a enfermidades graves que evoluem com freqüência para o óbito, ou causam seqüelas importantes, principalmente a deficiência mental.

**OBJETIVO:**

A possibilidade de detecção precoce de portadores motivou a triagem desses distúrbios entre recém-nascidos da Unidade Neonatal, na tentativa de iniciar o tratamento de suporte, quando disponível, antes que as manifestações clínicas fossem evidentes.

**TIPO DE ESTUDO:**

Estudo prospectivo.

**LOCAL:**

Unidade Neonatal em Hospital Terciário.

**PARTICIPANTES:**

101 crianças internadas na Unidade neonatal foram incluídas no estudo por apresentarem hipoglicemia, acidose metabólica, icterícia, dificuldade de ganhar peso, diarréia, vômitos, hepato e/ou esplenomegalia, catarata, apnéia, convulsões, hipo ou hipertonia.

**TESTES APLICADOS:**

Testes realizados para pesquisa qualitativa de substâncias anormalmente excretadas na urina em situações de desordem metabólica.

**RESULTADOS:**

Os recém-nascidos foram incluídos no estudo principalmente por apresentarem hipoglicemia, icterícia e acidose metabólica. 64 recém-nascidos apresentaram pelo menos um teste com resultado positivo. A positividade foi devida principalmente a distúrbios metabólicos transitórios, como por exemplo a Tirosinemia Transitória do RecémNascido, apresentada por 29 pacientes. Nove crianças foram selecionadas e encaminhadas ao Centro Medico de Genética para continuação da investigação diagnóstica. Para três delas os testes aplicados permitiram a formulação de uma hipótese diagnóstica de mucopolissacaridose, tirosinemia tipo I e hiperglicinemia não-cetótica, respectivamente.

**CONCLUSÕES:**

A alta positividade observada nos testes reflete a imaturidade metabólica própria do recém-nascido. A seleção de 9% dos casos triados para acompanhamento ambulatorial confirma que os erros inatos do metabolismo devem ser suspeitados sempre que um paciente apresente distúrbios metabólicos ou manifestações neurológicas sem causa determinada, sendo pesquisados paralelamente às demais possibilidades diagnósticas e não apenas tidos como diagnósticos de exceção.

## INTRODUCTION

Inborn Errors of Metabolism are hereditary affections resulting from incompetence in enzymatic reactions of intermediary metabolism, due both to deficiency in the enzyme involved and to its lower activity or stability. This blockage generally causes an increase in the precursor for the compromised metabolic stage and a lack of subsequent intermediaries or activation of alternative metabolic routes, leading to the production of substances normally not encountered in the organism. These errors may occur in the metabolism of all the organic compounds, both in synthesis and catabolism situations and in energy production, with a great number of diseases resulting from these metabolic disturbances.^1^ At present, several hundred hereditary metabolic disturbances are known, many of which correspond to grave illnesses that frequently evolve to death or cause important sequelae, especially mental deficiency.^2^

Considered individually, inborn errors of metabolism present very variable incidences. Considered as a whole, they present a cumulative frequency of 1:500 newborns^5^ and are readily encountered in medical practice.^3–5^ In spite of this fact, the clinical diversity that these diseases present makes it difficult to recognize them clinically, with specific diagnosis being extremely dependent on laboratory assistance.^6,7^

The organized study and systematized planning of the diagnostic conditions among possible inborn errors of metabolism carriers would appear to be very promising activities insofar as these may make it possible to prevent and control morbidity that not long ago was considered to be irremediable.^8^ Moreover, such studies open the way towards therapy, which could be very efficacious in some cases, with continual advances being incorporated,^9^ as well as making it viable to offer genetic counseling and assistance in the couple's reproductive prognosis.^7,10^ Within inborn errors of metabolism diagnosis programs, biochemical screening procedures are of fundamental importance, as these serve as a first indication of the general metabolic routes that the alterations can be involved in, and the more specific tests needed for proceeding with the evaluation.^11,12^

Thus, the possibility of early detection of carriers has motivated the screening for these disturbances among newborns at the Neonatal Unit of the Hospital São Paulo, in an attempt to initiate support treatment, when available, before clinical manifestations become evident.

## METHODS

This study was made at the Laboratory for inborn errors of metabolism at the Center for Medical Genetics of the Departments of Pediatrics and Morphology of the Universidade Federal de São Paulo/Escola Paulista de Medicina (UNIFESP/EPM). The sample for this study was made up by neonates born in the Hospital São Paulo during a 14-month period.

Children were included in this study if they fulfilled the following inclusion criteria: presence of clinical or asymptomatic hypoglycemia (glycemia less than 40 mg/dl), metabolic acidosis (fall of 0.15 in pH and fall of 10 mEq/l in bicarbonate), jaundice, difficulty in gaining weight (weight gain <10g over four consecutive days, despite a calorific offer > 100 cal/kg), diarrhea, vomiting, hepato- and/or splenomegaly, cataracts, apnea, convulsions, hypo- or hypertonia.

The neonatal population was classified according to the sex, gestational age, chronological age, and appropriateness of weight to gestational age. The gestational age was calculated using Naegele's rule^13^ and by Dubowitz's method,^14^ performed between 6 and 24 hours after birth. The gestational age determined by Dubowitz's method^14^ was considered when the difference between this and the gestational age calculated using Naegele's rule^13^ was greater than two weeks, or when the date of the last maternal menstruation was not known, or further, when the maternal information was imprecise.

To classify the gestational age, the World Health Organization criteria^15^ were used, and the newborns were considered to be full-term when their gestational age ranged between 37 completed weeks and 42 uncompleted weeks, pre-term when the gestational age was less than 37 weeks, and post-term when the gestational age was greater than or equal to 42 weeks.

To classify the newborns according to gestational age and birth weight, the Battaglia & Lubchenko^16^ intra-uterine growth curves were utilized. Newborns were considered to have appropriate weight for the gestational age when the weight was located between the 10^th^ and 90^th^ percentiles of this curve. They were considered to be large for the gestational age when the birth weight was above the 90^th^ percentile, and small for the gestational age when the weight was below the 10^th^ percentile.

The newborns included in the study were submitted to the tests routinely utilized in the Laboratory for Inborn Errors of Metabolism at the Center for Medical Genetics, based on qualitative research for abnormal substances excreted in the urine in situations of metabolic disorder, as described by Buist,^17^ Giogio & Luhby^18^ and Thomas,^19^ and aminoacid urine chromatography ^20^.

The results from the tests were analyzed in parallel with the clinical information available, and in accordance with the demographic data for the newborns obtained from consulting their medical records from the internment in the Neonatal Unit.

All tests with a positive result were repeated, using a new urine sample, as a form of follow-up for these newborns. In the same way, all the newborns that persisted with the clinical alterations cited in the inclusion criteria for the study, but presented normal results in the first tests performed, had their tests repeated.

## RESULTS

The sample studied was made up of 101 newborns selected from among the births occurring at the Neonatal Unit during a 14 month period, representing 6.8% of the total number of births during the period (n = 1477).

These newborns were predominantly male, premature and with weight appropriate to the gestational age (Table 1). They were included in the study mainly because of the development of hypoglycemia, jaundice or metabolic acidosis (Table 2) and the majority were submitted to screening tests in the first week of life (61.4%).

**Table 1:** Baseline characteristics of participants

*Characteristics*	*n (%)*
***Sex***	
*Male*	*60 (59.4%)*
*Female*	*41 (40.6%)*
***Gestational Age***	
*Pre-term*	*64 (63.3%)*
*Term*	*34 (33.7%)*
*Post-term*	*3 (3%)*
***Birth Weight***	
*Small*	*23 (22.8%)*
*Appropriate*	*72 (71.3%)*
*Large*	*6 (5.9%)*
***Clinical Signs (Number of events#)***	
*Hypoglycemia*	*62 (61.4%)*
*Jaundice*	*56 (55.4%)*
*Metabolic acidosis*	*51 (50.5%)*
*Respiratory disturbance*	*44 (43.6%)*
*Apnea*	*14 (5.3%)*
*Seizures*	*12 (13.9%)*
*Hepatomegaly*	*6 (5.9%)*
*Hypotonia*	*6 (5.9%)*
*Ocular alterations*	*5 (4.9%)*
*Coma*	*3 (2.9%)*
*Difficulty in gaining weight*	*3 (2.9%)*
*Vomiting*	*3 (2.9%)*
*Lethargy*	*1 (0.9%)*

***# Some patients presented more than one sign.***

**Table 2:** Positive results for each applied test and situations for which they are usualyusually positive

*Positive situation*	*Tests*	*Positive results*
*Tyrosinemia*	*Nitrosonaphthol*	*30 (29.7%)*
*Mucopolysaccharidosis*	*CTA Bromide*	*20 (19.8%)*
*Mucopolysaccharidosis*	*Toluidine Blue*	*1 (1.0%)*
*Amino aciduria*	*Chromatographic alterations*	*19 (18.8%)*
*Reducing substances, particularly sugar, as in galactosemia, fructosemia*	*Benedict*	*9 (8.9%)*
*Organic aciduria*	*2,4-dinitrophenyl-hydrazine*	*2 (2.0%)*
*Cystinuria-Homocystinuria*	*Cyanide-nitroprusside*	*1 (1.0%)*
*Methylmalonic aciduria*	*p-nitroaniline*	*-*
*Phenylketonuria, tyrosinemia, maple syrup urine disease, histidinemia, alkaptonuria*	*Ferric Chloride*	*-*

Of these newborns, 63.4% presented at least one positive test result. The individual results of the tests are shown in Table 3, along with the conditions for them to be positive.^17,18,21^

**Table 3:** Influence of gestational age and post-natal age on test positivity, expressed as number of newborns with positive tests

*Gestational age*	*Post-natal age*			
	*1 to 7 days*	*8 to 14 days*	*> 14 days*	*Total*
*Pre-term*	*26 (40.6%)*	*14 (21.9%)*	*5 (7.8%)*	*45 (70.3%)*
*Full and post-Term*	*13 (20.3%)*	*3 (4.7%)*	*3 (4.7%)*	*19 (29.7%)*
***Total***	***39 (60.9%)***	***17 (26.6%)***	***8 (12.5%)***	***64 (100.0%)***

***c ^2^= 0.63; (g.l. = 4); P = 0.89.***

Table 3 shows the characteristics of the newborns that may be related to the presentation of a positive result in the tests applied, compared using the chi-squared (c^2^) test.

The majority of the positive results were observed in premature patients (c^2^ = 3.55) and in those submitted to tests in the first week of life (c^2^ = 2.56), even though these differences were not statistically significant. The association of both conditions in the newborns with positive results was not significant (c^2^ = 0.63) (Table 3).

For three of the patients studied, the tests applied permitted us to formulate a diagnostic hypothesis with greater confidence, respectively of mucopolysaccharidosis, tyrosinemia type I and non-ketotic hyperglycinemia, as will be seen in the discussion, although these tests alone did not confirm the diagnosis.

Regarding two other infants referred onwards, the diagnostic hypotheses were made mainly from the clinical findings. and the family history. One of them, included in the study because of presenting convulsive crises and hepatosplenomegaly, whose screening tests that were all normal, had two deceased sisters whose clinical state had included convulsions, loss of learned skills and hepatic insufficiency, for whom a diagnostic hypothesis of Alpers’ Syndrome had been proposed. The second child mentioned, the daughter of consanguineous parents, presented cyanosis, vomiting, tremor (without presenting hypoglycemia), convulsions, apnea, bradycardia, hypotonia and microcephaly. This child also had screening tests with normal results and had a family history of two deceased brothers with the same clinical condition. For this child, a diagnostic hypothesis of pyruvate dehydrogenase complex deficiency was put forward.

The other four children referred all presented persistent metabolic acidosis, two of whom also presented convulsive crises. For one of these, a low protein diet was introduced during its internment in the Neonatal Unit, which reversed the metabolic acidosis condition. In none of these four patients did the screening tests permit the establishment of a diagnostic hypothesis.

## DISCUSSION

Among the 101 newborns studied, approximately 63% presented a positive result in at least one of the tests to which they were submitted. Of all the tests applied, three merit highlighting because of the large numbers of positive results.

The cetyltrimethyl-ammonium bromide test, utilized for the detection of glycosaminoglycans in the urine, was a test that presented a great number of false-positive results. It was positive in situations in which the newborn also presented a positive result for other substances not normally encountered in urine, such as reducing substances, or an altered pattern of amino acid excretion in the urine. For this reason, this test was utilized as an indicator for performing the toluidine blue test, a more specific test for the detection of glycosaminoglycans in the urine. With this latter test, only one positive result was found and in this case, there was an indication for the follow-up of the patient for investigation of mucopolysaccharidosis.

In addition to the cetyltrimethyl-ammonium bromide test, the nitrosonaphthol test (specific for the detection of tyrosine and an excess of its metabolites in urine) also presented large positivity (29.7%), which we did not, however, consider to be a false-positive result. We believe that these are related to the newborn's own metabolic immaturity, especially when compared to the positivity of this test in examinations performed routinely at the Laboratory for Inborn Errors of Metabolism at the Center for Medical Genetics, which is only 1.0% (N = 1000).

These positive tests would indicate cases of transitory neonatal tyrosinemia. This condition is believed to be the most common alteration in the metabolism of amino acids in human beings, with an incidence of 10% having been described among full-term newborns and between 30 and 50% among premature newborns.^22^ This condition does not have an exclusive genetic determination (late maturation of hydroxyphenylpyruvate dehydrogenase and tyrosine amino transferase),^26^ but is also related to prematurity, high protein ingestion during the first days of life (> 3 g of protein/kg weight), low birth weight, low ingestion of vitamin C (a cofactor in compromised metabolic routes), among other factors.^22–26^

Transitory Neonatal Tyrosinemia is considered to be a benign condition, although there is a lack of recent studies to confirm this information.^27^ In older studies, a reduction in motor activity of these newborns was observed in relation to normal controls paired by gestational and post-natal ages,^28^ lethargy,^25^ and an intellectual deficit in children assessed around the ages of 4 to 5 years,^29^ although another study did not show any neurological or developmental alteration in children with transitory neonatal tyrosinemia assessed at 2 years of age. Nevertheless, greater morbidity was observed among these children, especially related to the respiratory system.^30^

Recently, Jardim et al.^31^ evaluated children presenting transitory neonatal tyrosinemia at 4 years of age, observing slight intellectual deficits in these children in comparison with the control group. In this same study, a possible association of this condition with behavioral problems such as attention deficit and hyperactivity was suggested.

In our sample, the newborns that presented this condition were predominantly premature (80.0%), born with weight appropriate to the gestational age (70.0%), submitted to screening tests within the first two weeks of life (93.3%) and presented metabolic acidosis (73.3%), respiratory insufficiency (60.0%), hypoglycemia (56.7%), jaundice (53.3%) and sepsis (46.7%) during hospital internment.

When they presented a positive result in the nitrosonaphthol test, the newborns received oral supplements of vitamin C (100 mg/day)^22^ and had their test repeated after approximately one week, so that the transitoriness of the disturbance could be confirmed. Only one result continued to be positive after the repetition of the test, and the patient was referred to the Genetic Unit with a diagnostic hypothesis of tyrosinemia type I.

Regarding the alterations on aminoacid urine chromatograms, the majority of them were transitory alterations, except for one patient who continued with increased excretion in the glycine migration band, which is being followed up with a diagnostic hypothesis of non-ketotic hyperglycinemia.

As in the case of hypertyrosinemia, these transitory alterations are a result of the newborn's own immaturity, which is known to be capable of causing alterations in the excretion of various aminoacids.^32^ They have the same contributory factors, i.e. prematurity, low weight, the quantity and quality of the diet, the rate of growth and renal immaturity.^33,34^

Although we selected 9% of the patients for outpatient follow-up, these patients are still proceeding with investigations to confirm the diagnosis. Our percentage of selected individuals for further investigation is greater than that found in other studies made on selected populations in Brazil, which have shown inborn errors of metabolism detection rates of between 6.5 and 7.7%.^35–38^ On the other hand, a more recent work from Sanseverino et al.^11^ detected a 13% rate of inborn errors of metabolism in patients from pediatric and neonatal intensive care units with more similarities to the group of patients we studied.

## CONCLUSION

The high positivity observed in the screening tests is of fundamental importance for directing the clinical investigation, especially in places where more specific examinations are unavailable. It depends on an intimate association between the interpretation of the biochemical tests and a specialized multi-disciplinary clinical investigation.

The selection of 9% of the screened cases for outpatient follow-up confirms that inborn errors of metabolism must be suspected whenever a patient presents metabolic disturbances or neurological manifestations without a determined cause. They should be researched in parallel with the other diagnostic possibilities and not just taken to be exceptional diagnoses.
